# Lineage analysis of early and advanced tubular adenocarcinomas of the stomach: continuous or discontinuous?

**DOI:** 10.1186/1471-2407-10-311

**Published:** 2010-06-21

**Authors:** Takahisa Nakayama, Zhi-Qiang Ling, Ken-ichi Mukaisho, Takanori Hattori, Hiroyuki Sugihara

**Affiliations:** 1 Department of Pathology, Shiga University of Medical Science, Otsu, 520-2192 Japan; 2 Laboratory of Molecular Pathology, Zhejiang Cancer Research Institute, Zhejiang Province Cancer Hospital, Hangzhou, 310022, China

## Abstract

**Background:**

Eradication of early gastric carcinoma (GC) is thought to contribute to reduction in the mortality of GC, given that most of the early GCs progress to the advanced GCs. However, early GC is alternatively considered a dormant variant of GC, and it infrequently progresses to advanced GC. The aim of this study was to clarify the extent of overlap of genetic lineages between early and advanced tubular adenocarcinomas (TUBs) of the stomach.

**Methods:**

Immunohistochemical staining for p53 was performed using 28 surgically resected stomachs with 13 intramucosal and 15 invasive TUBs. By chromosome- and array-based comparative genomic hybridization (CGH), genomic copy number constitution was compared between the mucosal and invasive parts of the invasive TUBs and between the mucosal parts of the invasive and intramucosal TUBs, using 25 and 22 TUBs, respectively. *TP53 *mutation in exons 5-8 was examined in 20 TUBs.

**Results:**

Chromosomal CGH revealed that 4q+ and 11q+ were more common in advanced and early TUBs, respectively, whereas copy number changes in 8q and 17p showed no significant differences between early and advanced TUBs. However, array CGH revealed that, of the 13 intramucosal TUBs examined, loss of *MYC *(*MYC*-) and gain of *TP53 *(*TP53*+) was detected in 9 TUBs and *MYC*+ and/or *TP53*- was detected in 3 TUBs. Of the mucosal samples of 9 invasive TUBs, 7 showed *MYC*-/*TP53*+ and none showed *MYC*+ and/or *TP53*-. Of the 9 samples from the invasive parts, 1 (from submucosal cancers) showed *MYC*-/*TP53*+ and 6 (1 from submucosal and 5 from advanced cancers) showed *MYC*+ and/or *TP53*-. The latter 6 tumours commonly showed a mutant pattern (diffuse or null) in p53 immunohistochemistry, and 4 of the 6 tumours assessable for *TP53 *sequence analysis revealed mutations. The overall array CGH pattern indicated that, between the mucosal and invasive parts, genetic lineage was found discontinuous in 5 advanced cancers and continuous in 3 submucosal cancers.

**Conclusions:**

Genetic lineages often differed between early and advanced TUBs. *MYC*-/*TP53*+ and *MYC *+ and/or *TP53*- may be the signatures of dormant and aggressive TUBs, respectively, in the stomach.

## Background

Gastric cancer remains the second most common cause of cancer-related deaths worldwide, despite a recent decrease in its mortality in developed countries [1]. Gastric carcinomas (GCs) are classified morphologically into 2 major categories: tubular-forming type and diffuse type [2,3] and, in staging, into early cancers (involving the mucosa and the submucosa) and advanced cancers (involving the muscularis propria or deeper). Early GC is considered a curable cancer [4]; it reportedly progresses to advanced GC after varying durations as an early stage of GC [5,6]. In Japan, early GCs can be actively resected endoscopically as well as surgically [7], based on the principles of early detection and treatment. On the other hand, some pathologists maintain that the tubular-forming neoplastic lesions that are confined to the mucosa take a long time to or are unable to invade deeper tissues. Thereby, these lesions are called dysplasia [4].

Recently, a mass-screening program for neuroblastomas in Japan [8-10] was suspended because a discontinuous genetic lineage was found between the early- and the late-presenting neuroblastomas. Negative and late-presenting (≥1 year) neuroblastomas showed near-diploidy with loss of terminal 1p, whereas positive neuroblastomas in infants showed near-triploidy without 1p deletion [11,12]. On the other hand, comparative genomic hybridization (CGH)-based analysis suggested that high-grade atypical adenomatous hyperplasia in the lung is the true precursor of bronchioloalveolar carcinoma [13]. In the stomach, it is a problem to what extent genetic lineage is overlapped in early and advanced GCs.

In diffuse type GCs, we used CGH to demonstrate that chromosomal copy-number alterations in the intramucosal signet-ring cell carcinomas that showed a layered structure [14] were inherited in a fraction of poorly differentiated GCs at advanced stages [15]. The presence of a layered structure of signet ring cells in the mucosal parts of these advanced cancers also suggested that signet ring cell carcinomas may be a true precursor of poorly differentiated GCs. CGH-based lineage analysis of another subset of poorly differentiated advanced GCs with tubular components but no layered structure in the mucosal part revealed that GCs of this subset were derived from a tubular component in a tumour and were characterized by 17p- and 8q+ [16] and inactivation of the wild-type *TP53 *by mutation and loss of heterozygosity (LOH) [17]. In order to determine the early-stage counterpart of this type of GC, we analysed intramucosal tubular adenocarcinomas (TUBs) using CGH. These studies found that the intramucosal TUBs showed not only several chromosomal changes common to advanced GCs but also frequently showed 8q- and 17p + that were critically different from advanced and poorly differentiated GCs [18].

In the present study, copy-number changes of chromosomes and genes were examined in the mucosal and invasive parts of another series of early and advanced TUBs using array and chromosomal CGH. Based on these data, we demonstrated cases of continuous and discontinuous lineages between intramucosal and invasive parts of individual tumours and found that *TP53 *and *MYC *may be good lineage markers for gastric TUBs.

## Methods

The Institutional Review Board on Medical Ethics at Shiga University of Medical Science granted approval for conducting this research on the condition that the materials used must be anonymous.

### Tumour samples

This study included 28 surgically resected stomachs (Table 1) with 13 intramucosal TUBs and 15 invasive TUBs [6 submucosal and 9 advanced cancers that invaded the muscularis propria or deeper tissues (the tubular component of each tumour was assessed to be more than 30%)]. Each stomach was fixed in formalin and embedded in paraffin wax. These were selected at random from the materials diagnosed in our department from 1996 to 2008. Histological types and tumour stages were determined according to the Japanese Classification of Gastric Cancer [19] and pTNM staging, respectively. When a mucosal histological pattern was found to be heterogeneous in an individual invasive tumour, the part with the lowest grade of atypism was taken as the mucosal sample that can prevent the possibility of re-invasion of invasive cancer cells into the mucosa. In invasive cancers, DNA samples were obtained from intramucosal and invasive parts.

**Table 1:** Summary of clinical, histopathological and molecular genetic data of 25 tubular adenocarcinoma of stomach

Case No.	Age/sex	Histological type*	Invasion**	LN**	Stage **	CGH	p53 IHC	*TP53 *mutation
M1	57/M	tub1	T1(M)	N0	IA	c/a	+	NT
M2	72/M	tub1	T1(M)	N0	IA	c/a	+	NT
M3	67/F	tub1	T1(M)	N0	IA	c/a	-	-
M4	67/M	tub1	T1(M)	N0	IA	c/a	-	-
M5	74/M	tub1	T1(M)	N0	IA	c/a	+	-
M6	65/M	tub1	T1(M)	N0	IA	c/a	+	-
M7	67/F	tub1	T1(M)	N0	IA	c/a	+	-
M8	70/F	tub1 > pap	T1(M)	N0	IA	c/a	-	-
M9	51/M	tub1 > tub2	T1(M)	N0	IA	c/a	+	-
M10	52/M	tub2	T1(M)	N0	IA	c/a	+	-
M11	57/F	tub2	T1(M)	N0	IA	NT/a	-	-
M12	89/M	tub2	T1(M)	N0	IA	NT/a	+	+
M13	70/M	tub1	T1(M)	N0	IA	NT/a	-	NA
S1m	73/F	tub2	T1(SM)	N0	IA	c/a	+	-
S1i						NT/a	+	-
S2m	81/F	pap	T1(SM)	N1	IB	c/a	+	-
S2i						NT/a	+	+
S3m	79/M	tub1	T1(SM)	N0	IA	c/a	+	+
S3i						NT/a	+	+
S4m	63/M	tub2 > tub1	T1(SM)	N0	IA	c/NT	+	NT
S5m	75/M	pap > tub2	T1(SM)	N1	IB	c/NT	+	NT
S6m	79/M	pap	T1(SM)	N1	IB	c/NT	+	NT
A1m	71/M	tub2	T2(SS)	N2	IIIA	c/a	null	-
A1i						NT/a	null	NA
A2m	73/M	tub1 > tub2	T2(SS)	N0	IB	c/a	+	-
A2i						NT/a	+	+
A3m	56/M	pap	T2(SS)	N1	II	c/a	+	-
A3i						NT/a	+	+
A4m	81/M	tub2/pap	T2(SS)	N2	IIIA	c/a	null	-
A4i						NT/a	null	+
A5m	56/M	tub1	T2(MP)	N0	IB	c/NT	-	NT
A5i						NT/NA	-	NA
A6m	45/F	tub2	T2(SS)	N1	IB	c/NT	+	NT
A7m	79/F	tub2	T2(MP)	N1	IB	c/NT	+/-	NT
A8m	69/M	tub1	T3(SE)	N2	IIIB	c/a	null	-
A8i						NT/a	null	NA
A9m	67/F	tub2	T2(SS)	N1	II	c/a	+	-
A9i						NT/a	+	+

* Japanese classification of gastric carcinoma; ** pTNM classification. In the column of Case No, M = intramucosal cancer; S = cancer involving the submucosa; A = advanced cancer; m = mucosal part; i = invasive part. In the column of CGH, c = chromosomal CGH; a = array CGH. In the column of p53 immunohistochemistry (IHC); "+" indicates diffusely (>70%) positive nuclei, "-" indicates sporadically (<5%) positive nuclei; "null" indicates no immunoreactivity at all. In the column of *TP53 *mutation; "+" and "-" indicate presence and absence of mutation, respectively; NA = not assessable; NT = not tested.

### Immunohistochemistry

Immunohistochemical staining was performed with monoclonal antibodies to p53 protein (DO-7, 1:100; Dako, Glostrup, Denmark). After antigen retrieval of tissue sections in distilled water at 121°C for 5 min, immunoreactivity was detected by an indirect biotin-streptavidin-peroxidase method using the Histofine Kit (Nichirei, Tokyo, Japan) and diaminobenzidine reaction. The sections were counterstained with haematoxylin. Slides of the negative control without the primary antibody and those of the positive control were processed in parallel.

### Laser microdissection and DNA preparation

Tumour cells were obtained from 5-μm-thick tissue sections using a LMD6000 laser microdissection system (Leica Microsystems, Wetzlar, Germany). For individual tumours, cancer cells were obtained from areas >3 mm^2^, where cancer cells accounted for ≥90% of the total cell count. These cancer cells were digested in 200 μl of proteinase K solution at a concentration of 200 μg/ml for approximately 70 h at 37°C, followed by phenol/chloroform DNA extraction.

### Whole genome amplification

Sample DNA was amplified using degenerate oligonucleotide-primed polymerase chain reaction (DOP-PCR) in 2 phases, as described previously [20], which resulted in PCR products more than 2 kb in size, suitable for nick-translation labelling for CGH.

For array CGH, sample DNA was amplified using the GenomePlex Tissue Whole Genome Amplification Kit (WGA2 Kit; Sigma, St. Louis, USA) [21]. For some DNA samples that could not be sufficiently amplified, the WGA5 Kit (Sigma) was employed.

### CGH (hybridization, probe DNA labelling and digital image analysis)

DOP-PCR-amplified tumour and normal DNA was labelled using fluorescein-12-dUTP and tetramethylrhodamine-5-dUTP (Roche, Mannheim, Germany), respectively, by nick translation [15]. Hybridization and image analyses were performed as described previously [22]. Gains and losses in DNA copy numbers were defined by green to red ratios >1.2 and <0.8, respectively. Chromosomes 1p32-pter, 16p, 19, 22 and Y were excluded from these analyses.

### Array CGH

Oligo CGH microarray (60 K, 60-mer) (Agilent, Santa Clara, USA) was used in this study, according to the manufacturer's instructions. In brief, tumour and control DNA was non-enzymatically labelled with Cy5 and Cy3, respectively, using the Genome DNA ULS Labelling Kit (Agilent) and competitively hybridized to the microarray. Using Feature Extraction Ver.9.5.3 (Agilent), the fluorescence intensity of the tumours and controls was calculated from the hybridized array images captured using a DNA microarray scanner (Agilent). Copy-number gains and losses were defined as base 2 logarithm of the tumour signal intensity to the reference signal intensity ratio more than 0.3219 and less than -0.3219, respectively.

### Mutation analysis for TP53

PCR primer sets were prepared for exons 5-8 of *TP53*, which hybridize to the flanking introns of each exon. See additional file 1 for primer sequences. The first PCR mixture consisted of a buffer, 200 μM dNTPs, 1.5 mM MgCl_2_, 0.8 μM primer, 1 ng/μl sample DNA and 0.5 U Platinum Taq (Invitrogen, California, USA) in a 25-μl final volume. After initial denaturation at 94°C for 2 min, 40 cycles of PCR were performed at 94°C for 30 sec, 54°C for 1 min and 72°C for 1 min, followed by the final extension step at 72°C for 10 min. After confirmation of PCR products by agarose gel electrophoresis, sequence PCR was performed using the BigDye Terminators v1.1 Cycle Sequencing Kit (Applied Biosystems, California, USA). The reaction mixture consisted of a buffer, 2 μl of the first PCR product, 0.15 μM primer and 0.5 μl BigDye Terminator V 1.1 in a 10-μl final volume. After initial denaturation at 96°C for 1 min, 25 cycles of PCR were performed at 96°C for 10 sec, 50°C for 5 sec and 60°C for 4 min. The forward and reverse sequences were determined using the ABI PRISM 3100 Genetic Analyser (Applied Biosystems). Mutations were detected by comparing these samples to the reference DNA sequence (GenBank accession number: HSU94788). Allelic status was determined using the ratio of mutant to wild-type peak levels in sequencing profiles.

### Statistical analyses

Contingency tables were analysed by Fisher's exact test and Cochran-Armitage tests. Differences of 2-sided tests with P < 0.05 were considered to be statistically significant.

## Results

### Immunohistochemistry for p53

Staining patterns were considered overexpressed only when ≥70% tumour cells showed positively stained nuclei when viewed in a low-power field. Eight of the 13 intramucosal cancers, all the 6 submucosal cancers and 4 of the 9 advanced cancers showed diffusely positive nuclear staining for p53 (Table 1). A focal staining pattern was observed in case #A7. A pattern was considered negative (representing the wild-type *TP53*) when the presence of sparse, sporadic and weak nuclear staining was observed in <5% of the nuclei. A null staining pattern was observed in cases #A1, #A4 and #A8, suggesting a non-sense mutation of *TP53 *[23].

### Chromosomal CGH

Twenty-five samples from 25 cases (10 mucosal, 6 submucosal and 9 advanced cancers) were used for chromosomal CGH. All these samples were obtained from intramucosal lesions that showed the lowest grade of atypism.

While 4q+ occurred in the majority (6/9 cases) of advanced TUBs, it was not detected in the 16 early TUBs (P = 0.0005). Another significantly different chromosomal change between early and advanced cancers was 11q+, which was detected in 11 of the 16 early cancers and 2 of the 9 advanced cancers (P = 0.0414). Of the 10 intramucosal TUBs examined, 3 showed loss of 8q (8q-) and/or gain of 17p (17p+), while 7 and 2 showed 8q+ and 17p-, respectively. Of the 15 mucosal samples of invasive TUBs examined, 3 and 4 showed 8q- and 17p+, respectively, while 7 and 6 showed 8q+ and 17p-, respectively.

### Array CGH

**Figure 1:**
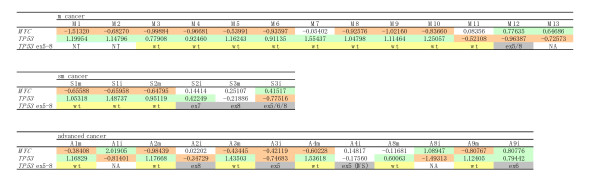
**Array CGH data of *MYC *and *TP53 *and *TP53 *sequencing data in early and advanced tubular adenocarcinomas of stomach**. Early cancers are divided into intramucosal (m) cancers and submucosal (sm) cancers. Refer to Table 1 for sample numbers. Numerals are mean test/reference ratios of array CGH. Significant losses and gains are indicated with red and green, respectively. Wild type (wt) and mutation in exon (ex) and intervening sequence (IVS) of *TP53 *are indicated with yellow and gray, respectively.

Thirty-one samples from 22 cases (13 mucosal, 3 submucosal and 6 advanced cancers) were assessed using array CGH (Figure 1). DNA samples were obtained from intramucosal and/or invasive lesions.

As shown in Figure 1 and Table 2, concomitant loss of *MYC *(*MYC*-) and gain of *TP53 *(*TP53+*) were detected in 9 of the 13 intramucosal TUBs, 7 of the 9 mucosal samples of invasive TUBs, 1 of the 3 invasive samples of submucosal cancers and none of the 6 invasive samples of advanced cancers. A pattern of *MYC+ *and/or *TP53*- was detected in 3 of the 13 intramucosal cancers, none of the 9 mucosal samples of invasive TUBs, 1 of the 3 invasive samples of 3 submucosal cancers and 5 of the 6 invasive samples of 6 advanced cancers.

**Table 2:** Alterations of *MYC *and *TP53 *copy numbers in early and advanced tubular adenocarcinomas of stomach

Tumour depth	Mucosa		Submucosa			MP or deeper -		
			**Mucosal part**		**SM**	**Mucosal part**		**Deep**

	cCGH (10)	aCGH (13)	cCGH (6)	aCGH (3)	aCGH (3)	cCGH (9)	aCGH (6)	aCGH (6)

8q(*MYC*)+	7	2 (2*)	3	**0**	1 (1*)	4	**0**	3 (2*)

8q(*MYC*)-	3	9 (9**)	3	2 (2**)	1 (1**)	0	5 (5**)	1 (0**)

17p(*TP53*)+	3	10(9**)	3	2 (2**)	2 (1**)	1	6 (5**)	1 (0**)

17p(*TP53*)-	2	3 (2*)	2	**0**	1 (1*)	4	**0**	4 (2*)

SM, the submucosa; MP, the muscularis propria. cCGH and aCGH, chromosome- and array-based comparative genomic hybridisation; Numerals in parentheses indicate the total number of the samples examined. Bold letters indicates the results of aCGH. Asterisks in parentheses indicate concomitance of *MYC*+ and *TP53*-. Double asterisks indicate concomitance of *MYC*- and *TP53*+.

**Figure 2:**
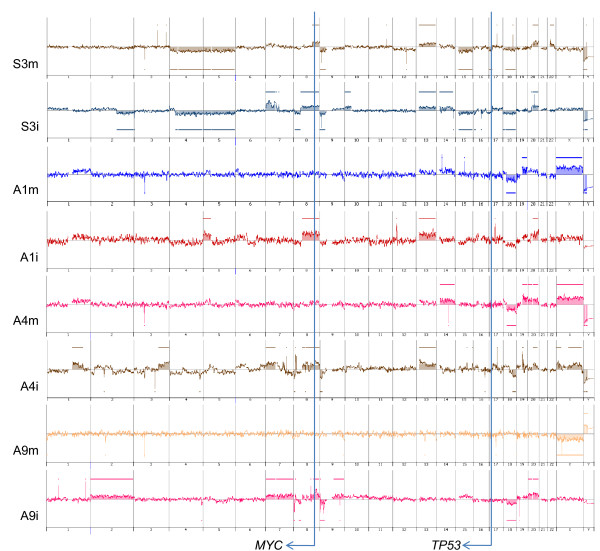
**Array CGH profiles**. The chromosomal regions of copy number aberrations in the mucosal part of #S3 (S3m) are included basically in those in the invasive part (S3i), whereas those in the mucosal part of #A1 (A1m) are different from those of the invasive part (A1i).

Based on the overall array CGH patterns, genetic lineages were found to be discontinuous and continuous between the mucosal and invasive parts in 5 advanced and 3 submucosal cancers, respectively. As shown in Figure 2, the sample from the mucosal part of #S3 showed 4p-, 4q-, 8q+, 9p-, 13q+, 15q-, 18q-, 20q+ and Y- , while the sample from the invasive part of #S3 inherited the same aberrations from the mucosal part, showed additional aberrations (2q-, 7p+, 8p-, 10p+, 14q+ and 20q+) and lost 4p- and 22q+. In #S1, gains of 1q, 14q, 19 and 20, and loss of 18q was common in mucosal and submucosal samples, though the gains of 1q, 14q, 19q and 20 in the submucosa were slightly less than the significant level. In #S2, a common change in both the mucosal and submucosal parts was a gain of 20p. The submucosal sample showed additional gains in 10p, 12 and 13q and losses in 3q, 4, 5, 8p, 9p, 9q, 10q, 12p, 14q, 16 and 19p. The mucosal sample showed loss of 18q, which was restored in the submucosal sample, possibly by uniparental disomy [24,25]. In #A1, #A3, #A4, #A8 and #A9, aberrations in the samples from the mucosal parts were not detected in those of the invasive parts. In #A2, the continuity of genetic lineages could not be assessed because of the absence of significant copy-number alterations in the mucosal part.

### *TP53 *mutation analysis

**Figure 3:**
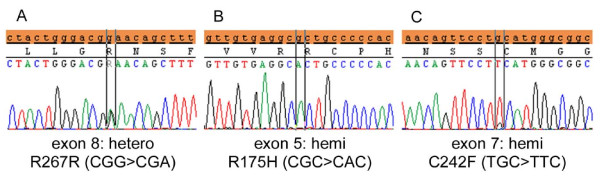
***TP53 *mutation patterns**. A heterozygous mutation with a significant wild-type component in exon 8 (A) and a hemizygous mutation in exon 5 (B) in an intramucosal cancer (#M12). Mutations in the other tumours examined were hemizygous as shown in C (#S2).

**Figure 4:**
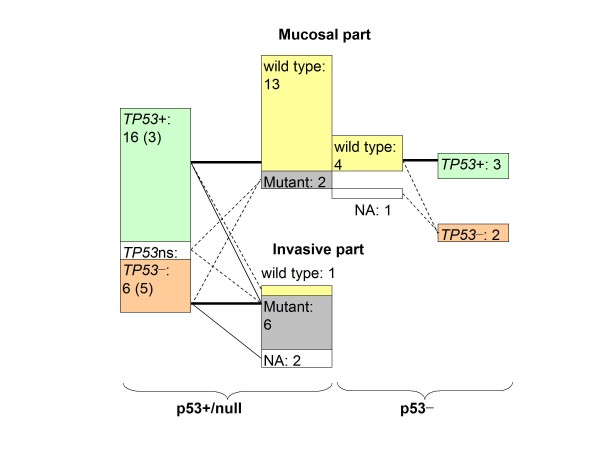
**Relationships among p53 immunohistochemistry, copy number of *TP53 *and hot spot mutation of *TP53***. For "p53+" and "p53-", see the legend in Table 1. "*TP53 *+", "*TP53 *-" and "*TP53 *n/s" indicate significant copy-number gain and loss, and no significant change, respectively. Numerals in parentheses indicate the number of samples in invasive parts. Numerals are sample numbers. Numerals in parentheses indicate the number of samples in invasive parts. NA = not assessable.

Mutations were detected in 7 of the tumours that were examined for sequence analysis of exons 5-8 of *TP53 *(Figure 1, Table 3): 1 of the 10 intramucosal cancers, 2 of the 3 submucosal cancers and all 4 advanced cancers (only the invasive parts). In all these 7 tumours, the *TP53 *allele was hemizygous except in 1 intramucosal cancer (#M12) with a heterozygous pattern for an exon-8 mutation (Figure 3) and a diffuse or a null pattern for p53, as indicated by immunohistochemistry. No *TP53 *mutation was detected in p53- samples, as shown in Figure 4. In p53+/null samples, 2 of the 15 samples of the mucosal part showed *TP53 *mutation, whereas 6 of the 7 samples of invasive part showed *TP53 *mutation (P = 0.0023). In 1 of the submucosal cancers showing *TP53*+ and a hemizygous mutation (the invasive part of #S2), *TP53*+ may reflect uniparental polysomy [24,25].

**Table 3:** Copy number changes of *TP53 *and *MYC *and p53 immunoreactivity in the samples tested for mutation analyses

Case No.	*MYC*	*TP53*	Mutation of *TP53 *exons 5-8	Mutant allele	*MDM*2	p53 IHC
M3	-	+	wt		-	-
M4	-	+	wt		ns	-
M5	-	+	wt		ns	+
M6	-	+	wt		-	+
M7	ns	+	wt		-	+
M8	-	+	wt		ns	-
M9	-	+	wt		ns	+
M10	-	+	wt		-	+
M11	ns	-	wt		ns	-
M12	+	-	Exons 5/8	hemi/hetero	ns	+
M13	+	-	NA		ns	-
S1m	-	+	wt		-	+
S1i	-	+	wt		-	+
S2m	-	+	wt		-	+
S2i	ns	+	exon 7	hemi	ns	+
S3m	ns	ns	exon 8	hemi	ns	+
S3i	+	-	exons 5/6/8	hemi	ns	+
A1m	-	+	wt		-	null
A1i	+	-	NA		-	null
A2m	-	+	wt		ns	+
A2i	+	-	exon 8	hemi	ns	+
A3m	-	+	wt		ns	+
A3i	-	-	exon 5	hemi	-	+
A4m	-	+	wt		-	null
A4i	ns	ns	exon 5	hemi	ns	null
A8m	ns	+	wt		-	null
A8i	+	-	NA		ns	null
A9m	-	+	wt		-	+
A9i	+	+	exon 6	hemi	ns	+

ns = not significant; wt = wild type; NA = not assessable; hemi = hemizygous; hetero = heterozygous.

M12, exon 5: R175H (CGC > CAC), C182C (TGC > TGT), S183L (TCA > TTA); exon 8: R267R (CGG > CGA)

S2i, exon 7: C242F (TGC > TTC)

S3m, exon 8: R273S (CGT > AGT)

S3i, exon 5: A161V (GCC > GTC); exon 6: T211I (ACT > ATT); exon 8: R273S (CGT > AGT)

A2i, exon 8: R273 H (CGT > CAT)

A3i, exon 5: A161T (GCC > ACC)

A4i, exon 5: IVS5+1G > T

A9i, exon 6: Y220C (TAT > TGT)

On the other hand, in the mucosal samples, the diffuse/null pattern was often associated with wild-type *TP53*. In order to explain this finding, the *MDM2 *copy number was examined using array CGH data. Of a total of 22 samples assessed for *TP53 *mutation analysis, *MDM2 *showed copy-number loss in 10 of the 14 samples with wild-type *TP53 *and 1 of the 8 samples with *TP53 *mutation (P = 0.0237).

## Discussion

In intramucosal TUBs, chromosomal CGH revealed 8q- and/or 17p+ at a frequency of 3 out of 10 tumours examined; these changes were more common in a previous study that used random priming labelling [18]. In the present study, 8q+ was frequently detected both in intramucosal cancers (7/10) and in invasive cancers (7/15); however, the above-mentioned previous data rarely showed 8q+. These discrepancies may reflect differences in the labelling methods used; CGH results with nick translation labelling or random priming labelling were compared to FISH signals, and it was found that nick translation labelling not infrequently showed false-positive gains [26]. However, using the same labelling conditions, significant differences in the incidence of 4q+ and 11q+ were detected between early and advanced cancers, possibly reflecting differences in genetic lineages between them. Chromosomal CGH data of advanced cancers in this study were consistent with previous chromosomal CGH data obtained with nick translation labelling for advanced GCs (i.e. 17p- and 8q+ were quite common) [27-30].

Although clinically discernible early cancers are generally considered the precursors of advanced cancers [5,6], the intramucosal and invasive parts of advanced TUBs often showed discordant copy numbers of *MYC *and *TP53 *in this study: *MYC*- and *TP53*+ in 5 of the 6 samples of the mucosal parts, as commonly as in the intramucosal cancers examined, and *MYC*+ and/or *TP53*- in 5 of the 6 samples of the invasive parts. In addition, based on the overall array CGH pattern of the mucosal and invasive parts of tumours, a discontinuous genetic lineage was found in 5 of the 6 advanced cancers. Therefore, it appears that the tumour clones in the mucosal parts of the advanced cancers were not precursors of the invasive part, but minute intramucosal cancers that probably coexisted with the invasive cancers. It has been reported that multiple minute cancers are more common in TUBs than in undifferentiated-type GCs [31]. In advanced TUBs, the intramucosal precursor of an invasive component might have been lost due to ulceration in the tumour, or the precursor was not examined because it did not coincide with the part of the lowest grade of atypism, to which the examination of the mucosal part of invasive GCs was confined in the present study.

On the other hand, in submucosal cancers, a continuous genetic lineage was found in all the 3 submucosal tumours examined. It may be because the mucosal part is basically retained in early GCs and because the mucosal precursor of an invasive component showed the lowest grade of atypism and was not excluded from the present array analyses. The mucosal and invasive parts had common regions of copy number aberrations with concordant breakpoints, and most of the changes in the mucosal part were included in those of the invasive part. At the gene level, the copy numbers of *MYC *and *TP53 *were consistent between the mucosal and submucosal parts in 2 of the 3 submucosal cancers.

The *TP53*- and/or *MYC*+ pattern was detected not only in the invasive parts of 1 of the submucosal cancers and most of the advanced cancers but also in 3 of the 13 intramucosal cancers. In these tumours, p53 immunohistochemistry showed a diffuse or null (mutation) pattern (Tables 1 and 3), suggesting that the *TP53*- may reflect LOH and the resultant inactivation of wild-type *TP53*. Sequencing analyses of the mutation hot spots of *TP53 *have demonstrated that hemizygous mutations (i.e. mutation and LOH) were detected in the mucosal samples of 1 of the 3 intramucosal cancers and 1 submucosal cancer with a *TP53*- pattern, as well as the invasive parts of the 4 advanced cancers that were informative for *TP53 *mutation analysis. Such inactivation of wild-type *TP53*, which may cause further genomic instability, has been frequently reported in advanced GCs [15,17]. The *MYC *gene locus is also known to frequently show copy-number gains or amplifications in various advanced cancers [32]. *MYC*+/*TP53*- may thus be justified as the signature of aggressive GC.

It has been reported that LOH as well as mutations of *TP53 *are more frequently detected in advanced GCs than in early GCs [17,33], and that, within early GCs, *TP53 *mutations are more frequently detected in submucosal TUBs than in intramucosal TUBs [34,35]. This tendency was also observed in the present study, but mutation analyses indicated that hot spot mutations were not detected in the mucosal part of the tumours, except in 1 intramucosal cancer (#M12). In addition, copy-number loss of *MDM2*, which plays a role in p53 degradation [36], correlated with diffuse p53 overexpression in the absence of a *TP53 *mutation. This phenomenon is scarcely reported, but could be one of the mechanisms of p53 overexpression in early GCs.

In early cancers, there may be dormant and invasive components as well as true precursors of advanced cancers. Among the early cancers examined in the present study, an invasive part of 1 tumour (#S3) and 3 intramucosal cancers (#M11, #M12 and #M13) showed the signature of aggressive cancer (*MYC*+ and/or *TP53*-) and frequent mutations of *TP53*; however, in 1 submucosal cancer (#S1), the invasive parts showed the signature of dormant cancer (*MYC*-/*TP53*+). Submucosal cancers with the signature of dormant cancer may be in the submucosa for a long time, and therefore, they provide a greater opportunity for clinical detection as submucosal cancers. However, in order to eradicate advanced cancers, early cancers with the signature of aggressive cancer must be detected, such as tumours #S3 and #M11-#M13. If this hypothesis of dormant and aggressive signatures is confirmed by studies with a larger number of cases, the differentiation between the 2 signatures can be applied to gastric biopsy specimens.

## Conclusions

These array CGH-based analyses have demonstrated that the copy numbers of *MYC *and *TP53 *have high discriminative power for differentiation between genetic lineages in individual gastric TUBs. This study has shown that genetic lineages are often different between early and advanced TUBs and that *MYC*-/*TP53*+ and *MYC*+ and/or *TP53*- may be signatures of dormant and aggressive TUBs, respectively, in the stomach.

## Competing interests

The authors declare that they have no competing interests.

## Authors' contributions

NT performed most of the experiments, participated in concrete research designing and most of the data analyses and drafted the manuscript. LZQ participated in parts of experiments, in particular, chromosomal CGH. MK participated in the preparation of samples in array CGH. HT provided critical comments and suggested revisions of the manuscript. SH was a leader of this project, who conceived the study, designed the research route, and guided the experiments and the data analyses. All authors read and approved the final manuscript.

## Pre-publication history

The pre-publication history for this paper can be accessed here:

http://www.biomedcentral.com/1471-2407/10/311/prepub

## Supplementary Material

Additional file 1 **TP53_primer_sequences **Microsoft Excel 2003 *TP53 *primer sequences. Click here for file
